# Micro/nano-bubble-assisted ultrasound to enhance the EPR effect and potential theranostic applications

**DOI:** 10.7150/thno.37593

**Published:** 2020-01-01

**Authors:** Lei Duan, Li Yang, Juan Jin, Fang Yang, Dong Liu, Ke Hu, Qinxin Wang, Yuanbin Yue, Ning Gu

**Affiliations:** 1 School of Biomedical Engineering and Informatics, Nanjing Medical University, Nanjing, 211166, P. R. China.; 2 State Key Laboratory of Bioelectronics, Jiangsu Key Laboratory for Biomaterials and Devices, School of Biological Science and Medical Engineering, Southeast University, Nanjing 210096, P. R. China.; 3 West Anhui University, Lu'an, P.R. China; 4 Anhui Engineering Laboratory for Conservation and Sustainable Utilization of Traditional Chinese Medicine Resources, P. R. China.

**Keywords:** EPR effect, ultrasound, micro/nanobubbles, controlled drug delivery, tumor theranostics

## Abstract

Drug delivery for tumor theranostics involves the extensive use of the enhanced permeability and retention (EPR) effect. Previously, various types of nanomedicines have been demonstrated to accumulate in solid tumors via the EPR effect. However, EPR is a highly variable phenomenon because of tumor heterogeneity, resulting in low drug delivery efficacy in clinical trials. Because ultrasonication using micro/nanobubbles as contrast agents can disrupt blood vessels and enhance the specific delivery of drugs, it is an effective approach to improve the EPR effect for the passive targeting of tumors. In this review, the basic thermal effect, acoustic streaming, and cavitation mechanisms of ultrasound, which are characteristics that can be utilized to enhance the EPR effect, are briefly introduced. Second, micro/nanobubble-enhanced ultrasound imaging is discussed to understand the validity and variability of the EPR effect. Third, because the tumor microenvironment is complicated owing to elevated interstitial fluid pressure and the deregulated extracellular matrix components, which may be unfavorable for the EPR effect, few new trends in smart bubble drug delivery systems, which may improve the accuracy of EPR-mediated passive drug targeting, are summarized. Finally, the challenging and major concerns that should be considered in the next generation of micro/nanobubble-contrast-enhanced ultrasound theranostics for EPR-mediated passive drug targeting are also discussed.

## Introduction

Solid tumors exhibit a special microenvironment featuring tortuous vascularity, angiogenesis, and hypoxia 1-3. Abnormal tumor vasculature, high interstitial fluid pressure, growth-induced solid stresses. And solid stresses from the abnormal stromal matrix are the main limitations that prevent drugs from penetrating the solid tumor 4, 5. Therefore, the accurate and efficient delivery of theranostic agents into tumor lesions in a controlled manner is critical and remains a significant challenge till date. However, the absence of vascular supportive tissues in tumors results in the formation of leaky vessels with pores (200 nm to 1.2 μm in diameter) 6 and leads to poor lymphatic drainage, which is the structural basis of the enhanced permeability and retention (EPR) effect. The EPR effect is considered an effective method to achieve the passive targeting of tumor tissues 7, 8. In preclinical studies, targeted delivery systems based on EPR have demonstrated a remarkable improvement in anticancer efficacy when compared with the efficacy of traditional chemotherapeutic drugs 7-9.

However, numerous parameters (such as vascular permeability and mutation, tumor cell density, and tumor stromal heterogeneity) prevent the successful application of EPR-based theranostics in clinical applications because of tumor heterogeneity 10-15. Different patients with the same type of tumor, different types and sizes of tumor, and different developmental stages of the same tumor may show different EPR effects 16, 17. For example, hepatocellular carcinoma and renal cell carcinoma both have high vascular density and exhibit a strong EPR effect, whereas pancreatic and prostate cancers are characterized by low vascular density, resulting in a lower EPR effect 18. Metastatic tumors have a high degree of necrosis at the central site relative to the primary tumor because of the lack of blood vessels due to the decrease in the EPR effect. Therefore, accurately monitoring and evaluating the EPR conditions of different tumors is essential to formulate personalized EPR-mediated tumor treatment 19.

In recent years, various nanomaterials based on the EPR effect have been used because the sizes of these nanomaterials can be modulated according to the pore sizes in tumor blood vessels. Because different types of tumors have different sized gaps in the endothelial cells, a suitable nanoparticle size must be selected according to the type of tumor. Furthermore, the interception by biological barriers when nanomaterials enter the circulation system also needs to be considered. Particularly, nanoparticles should not be destroyed by the reticuloendothelial system (increased particle size) while maintaining the ability to penetrate the tumor (reduced particle size) 20, 21. Although the gaps and pores in the wall of tumor vessels may provide a pathway for the exudation of nanoparticles, the intermittence and disorder of the vascular structure reduce the blood flow and intravascular pressure. This may result in the reduction of macromolecular drugs or nanocarriers entering the stroma through the vascular gaps by diffusion 22. Without further increasing the movement of these drugs via diffusion, the efficiency of delivery will not be significantly improved.

**Figure 1:**
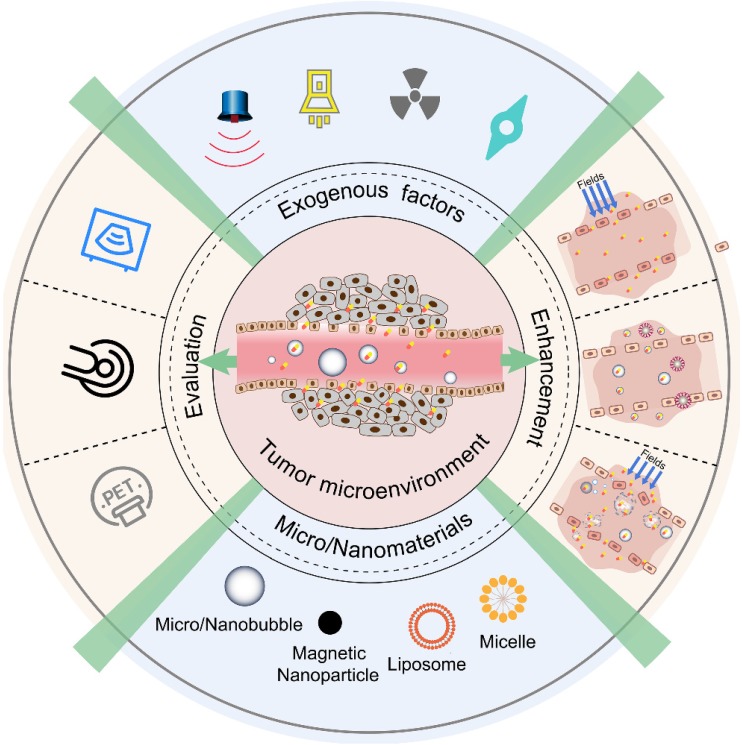
Various diagnostic and therapeutic strategies that make the best use of the EPR effect.

**Figure 2:**
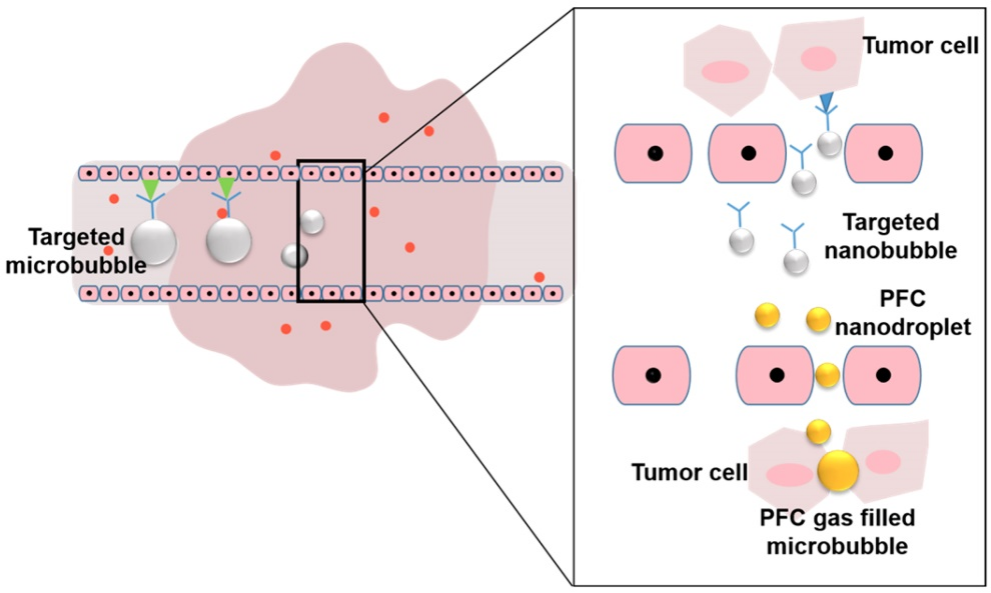
Transportation of micro/nanobubbles in the tumor tissue.

In view of these challenges, to best leverage the EPR effect in nanomedicine delivery, various diagnostic and therapeutic strategies have been developed (Figure 1). To monitor and evaluate the heterogeneity of the EPR effect in a particular patient, non-invasive imaging techniques (such as ultrasound, magnetic resonance imaging (MRI), and positron emission tomography (PET)) have been applied. With the development of contrast agents such as micro/nanobubbles (MNBs) 23-25, magnetic nanoparticles 26, 27, radionuclides, and fluorophores, the EPR effect can be accurately visualized and quantified 28-31. Nanomaterial-based EPR targeting strategies mainly focus on controlling the size of the drug or carrier and/or using ligands that bind to molecules that are related to the EPR effect. An appropriately sized material can pass through the permeable tumor vascular system and passively accumulate in the tumor tissue via passive targeting. The modification of the ligands on the surface leads to active targeting. The transport of the micro-materials and nanomaterials in tumor tissue is shown in Figure 2. Perfluorocarbon (PFC) nanodroplets can accumulate in the tumor tissue via passive targeting, and the targeted microbubbles and nanobubbles are used to realize active targeting.

To enhance the efficacy of delivery, three approaches based on the EPR effect have been developed. The first approach involves exerting physical exogenous factors (light 32-34, heat 35-37, ultrasound 38-41, and radiation 42, 43) to promote the permeability of tumor blood vessels and tissues. The second is to develop tumor microenvironment-targeted smart responsive nanomaterials to improve the permeability and retention of drugs at the tumor site 44-48. The third is to combine the internal biochemical response with external regulatory factors. Some micro-nano materials have special properties because of which they can respond to internal and external triggers. Using a series of biological effects generated during such a response, the drug delivery efficiency at the tumor site can be further improved. In this review, we summarize the EPR effects based on ultrasound and MNBs and the enhancement of these effects. First, the physical effects of ultrasound on EPR are briefly introduced. The interaction between EPR- and MNB-assisted acoustic energy is highlighted. Finally, some new trends in smart bubble drug delivery systems are summarized and speculated for future cancer theranostics.

## Ultrasonic effects and enhancement on EPR

Acoustic transducers are used to generate transverse or longitudinal waves with frequencies greater than 20 kHz. Medical ultrasound is characterized by a broad frequency range of 0.1-50 MHz and peak negative pressures of 0.01-10 MPa. It can be classified into focused and unfocused ultrasound depending on the radiation distribution of transducers. It can also be characterized into continuous and pulsed ultrasound depending on the waveform and with parameters such as pulse length and pulse repetition frequency. When used in biomedical applications, the acoustic densitometry of ultrasound can be expressed in the following manner. Sonic intensity can be defined as the time-averaged rate of sonic energy-flow through unit area 49. Ultrasound with a sonic intensity higher than 5 W/cm^2^ is generally referred to as high-intensity ultrasound, while that with an intensity between 0.5 and 1 W/cm^2^ is usually considered to be low-intensity ultrasound. In clinical ultrasound imaging, the output levels of the ultrasound equipment can be quantified using the mechanical index (MI) defined by the U.S. Food and Drug Administration (FDA). MI is expressed as the ratio of *in situ* peak negative pressure (PnP) to the square root of the center frequency (F_c_), as shown in Equation (1):


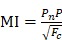
(1)

In general, the maximum output levels of diagnostic devices are limited to an MI of 1.9, which is the maximum allowed value for clinical imaging applications without microbubbles 50. Incompressible drug carriers such as micelles and liposomes can be applied with the maximum MI of 1.9, while compressible material such as microbubbles should be applied with the maximum allowable MI of 0.8 51.

When ultrasonic energy is applied for the diagnosis and treatment of tumors, acoustic waves can be used to enhance the EPR in two ways: drug release and bioeffects. During drug release, ultrasound can stimulate the carrier to release its cargo and increase the distribution concentration of drugs in the tumor. The efficiency of drug release is controlled by acoustic parameters such as ultrasound frequency, power density, and pulse duration 52, 53. Alexander et al. 54 compared the effects of continuous wave (CW) and pulsed ultrasound on doxorubicin (DOX) uptake by HL-60 cells. The drug uptake increased with a pulse duration in the range 0.2-2 s, and was comparable with CW ultrasound (10 s pulse) when a pulse with a duration of 2 s was applied. High-frequency ultrasound exhibits sharper focusing than low-frequency ultrasound, whereas low-frequency ultrasound penetrates the interior of the body deeper than high-frequency ultrasound. The typical penetration depth of 1-MHz ultrasound for various tissues is generally a few millimeters. In contrast, low-frequency ultrasound (20-100 kHz) can penetrate depths reaching tens of centimeters in some types of tissues. High-frequency ultrasound has, therefore, been advantageous for use in the targeted delivery of drugs to small superficial tumors, whereas low-frequency ultrasound is beneficial for treating large and deeply located tumors 55. For all frequencies studied 52, the drug release increases with increasing power density.

For bioeffects, the use of acoustic energy with EPR mainly focuses on the enhancement of cell membrane permeability 51. For example, Liu et al. 56 found that compared with other treatment intensities, ultrasonic exposure at 1 MHz and 0.25 W/cm^2^ can promote the platelet penetration of gold nanoparticles (GNPs). The results indicated that ultrasound can enhance membrane permeability, which has been proved using scanning electron microscopy (SEM) and transmission electron microscopy (TEM).

Besides, acoustic waves interact with drug carriers, body tissues, and cell membranes via a combination of thermal and mechanical effects 53. The key mechanisms by which EPR interacts with acoustic waves are mechanical and thermal energy. Low-intensity ultrasound (0.51 W/cm^2^) is known to be non-thermal, and this may be regarded as the boundary between mechanical and thermal effects.

### Mechanical effects

The mechanical effects of ultrasound and MNB-assisted ultrasound on EPR are based on both acoustic radiation forces and mechanical bioeffects.

#### Acoustic radiation force

Because of the momentum exchange between the object and the sound field, an acoustic wave can move suspended micro-objects by exerting a force known as the acoustic radiation force (ARF) 57.

The various forces that act on an air bubble in a sound field are often referred to as Bjerknes forces 58, which include two physical phenomena: primary Bjerknes forces and secondary Bjerknes forces. The force that influences the microcapsule *via* the ''primary'' (external) sound field is called the primary Bjerknes force and can be expressed as Equation (2). Assuming that the microcapsules are spherical and placed in an ideal plane ultrasonic wave, the force acts to propel the capsules in the direction of acoustic propagation as per the following equation:


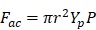
(2)

where *P* is the mean energy density of the incident wave, *Yp* is a dimensionless factor known as the radiation force function that depends on the scattering and absorption properties of the capsule, and *r* is the radius of the capsule.

The force between two bubbles that is caused by the ''secondary'' sound fields emitted by other bubbles is known as the secondary Bjerknes force 59. The secondary Bjerknes force is given by Equation (3):


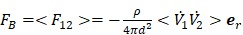
(3)

where < > denotes the time average, ρ is the density of the liquid, d is the distance between the first bubble and the second one, *V*_1_ and* V*_2_ describe the volume of the first and second bubbles, respectively, and **e***_r_* denotes the radial unit vector. The mutual interaction forces between two small coupled gas bubbles (R_0_<10 μm) in a strong low-frequency acoustic field (Pa>1 bar, f = 20 kHz) have been investigated in a previous study 59 using the Keller-Miksis equations. The nonlinear resonance of bubbles leads to secondary Bjerknes forces between the bubbles that are stronger by a factor of 10^3^-10^6^ than that expected from linear approximations.

The EPR effect would be considerably increased if the agent was initially concentrated in a particular region, and if the velocity of the agent passing the potential binding site was decreased. Dayton et al. 60 demonstrated that the radiation force of ultrasound can displace a microbubble contrast agent to the wall of a 50-mm blood vessel, significantly reduce the velocity of the flowing contrast agents, and produce reversible aggregation. Such microbubble aggregates in large vessels were observed optically under the influence of acoustic radiation force by Wang et al. 61. The first acoustic radiation force intravascular ultrasound transducer was designed to translate microbubbles to the vessel wall under flow velocities comparable with that in the human common carotid artery to enhance the binding efficiency of intravascular microbubbles and drug delivery 62.

#### Acoustic streaming

**Figure 3:**
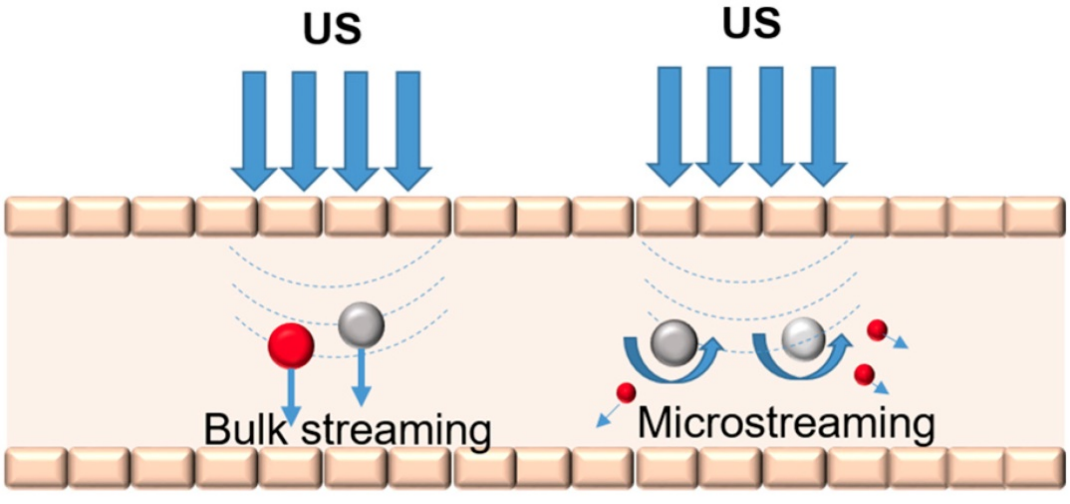
Bulk streaming and microstreaming in the blood vessel.

Acoustic streaming is defined as the radiation force from reflectors and scatterers in an ultrasonic field that can cause local particle displacements and fluid currents. The movement of localized fluid currents in the direction of the propagating ultrasound radiation forces that are induced by sound waves is termed bulk streaming. Microstreaming involves localized eddies or currents that are generated next to cavitating bodies. The differences between bulk streaming and microstreaming are shown in Figure 3. The acoustic streaming effect can be used to displace particles in the blood 63 or change the motion of blood flow 64. These mechanical actions generated by ultrasound may result in the release of drugs from carriers and the associated movement of the drug into the targeted tissue 65. Microstreaming with bubbles or particles plays an important role in *in vitro* low-frequency sonophoresis 66.

With the same acoustic-pressure amplitude, the maximum streaming velocity of ultrasound contrast agent (UCA) microbubbles is achieved under the action of resonance frequency. The velocity fields of the streaming sonicated UCA microbubbles were measured using particle image velocimetry (PIV) in a blood vessel model 67. The maximum streaming velocity was approximately 60 mm/s at a resonance frequency of 2.25 MHz, and the streaming velocity decreased to 15 mm/s at 1.0 MHz for the same amplitude of acoustic pressure.

#### Stable and inertial cavitation

Gas-filled bubbles show different oscillation performances when acoustic parameters such as the pulse length, amplitude, and frequency are varied. The stable cavitation (SC) of UCAs is usually accomplished at low sound pressures (<0.1 MPa). When exposed to sufficiently large acoustic rarefactional pressure amplitudes, UCAs undergo a violent collapse known as inertial cavitation (IC) 68. This phenomenon results in the generation of shock-waves, broadband acoustic emissions 69, jetting 70, and microstreaming 69. These effects have been identified as key mechanical triggers for various medical 40, 71 and industrial applications 72-74.

The difference between SC and IC is due to the different degrees of bubble deformation under ultrasonic irradiation. Many models have been developed to describe the deformations that can occur on the surface of a coated bubble because of oscillation. Marmottant et al. 75 presented a model that incorporates the effect of coating on the response of a microbubble to ultrasound. Based on the US-induced SC or IC, UCAs have wide applications in medical diagnosis and treatment such as contrast harmonic imaging and ultrasound-induced gene/drug delivery 76-78.

#### Mechanical-effect-enhanced EPR

For US-induced SC or IC of MNBs, MNBs are either excited, causing them to oscillate and mechanically massage the vascular wall, or are destroyed, generating micro-jets. These mechanical effects can lead to the creation of pores in the cell membrane. The opening of the inter-endothelial junctions contributes to an enhancement in the vessel permeability and improves the extravasation of co-administered drugs 79.

MNB-mediated sonoporation occurs because of the physical perturbation of cellular structures 80. Marmottant et al. 81 controlled vesicle deformation and lysis by a single oscillating bubble. They found that gentle bubble oscillations are sufficient to achieve the rupture of lipid membranes.

The enhanced efficiency can be evaluated through the accurate measurement of the spatial and temporal scales of these pores. Some methods are available to measure these pores. SEM and atomic force microscopy (AFM) have both been employed to obtain information about pores 82-85. The transmembrane current (TMC) of a single cell under a voltage clamp can also be used for monitoring sonoporation in real-time 86.

### Thermal effects

When a blood vessel is located near the focal spot, a part of the acoustic energy is absorbed and converted to thermal energy. Tissues may have different sensitivities to ultrasound-induced damage. This sensitivity to the effects of ultrasound-induced heating has primarily been studied by Stanley et al. 87. Thermal conversion efficiency is related to protein content and blood perfusion. For example, it has been reported that the absorption coefficient increases as a function of the protein content, which has a positive effect on the ultrasound-induced increase in temperature. Furthermore, biological tissue with poor blood perfusion can also be significantly heated by ultrasound because of slow heat dissipation 87.

#### Thermal-effect-enhanced EPR

Ultrasound energy can be absorbed and transformed into thermal energy in tissues as the acoustic waves propagate through the tissue and generate friction 51. Mild hyperthermia in a tumor (41-43 °C for 10-60 min) may improve the therapeutic efficacy of drugs by acting on tumor hemodynamics 88. The mild hyperthermia induced by focused ultrasound may, therefore, enhance drug or drug carrier extravasation in tumor tissue 65. In a previous study, a thermo-sensitive liposome was designed 89, and the results showed that drug release can be triggered by mild hyperthermia when the temperature is just above the normal physiological temperature.

Estimating the efficiency of the temperature rise caused by radiation of ultrasound is critical. When the distance between the surface of the blood vessel and the focal point is 2.5 mm, the temperature at the focal point was saturated after approximately 50 s of radiation, and the maximum cooling caused by the blood flow was approximately 0.5 °C when the supply of radiation was ceased 90. Based on the thermal wave model of bioheat transfer, Tan et al. numerically investigated the influence of blood vessels on temperature during high-intensity focused ultrasound hyperthermia 91. The results showed that a longer duration of thermal relaxation leads to the production of smaller thermal lesions.

## MNB-assisted ultrasonic evaluation and its enhancement on EPR

MNBs comprise a gaseous core encapsulated in a shell of biocompatible materials such as lipids or proteins, typically ranging from 0.1 to 10 μm in diameter. When exposed to ultrasound, MNBs can contract and expand, which may result in backscattering of the ultrasound, cavitation, or even bursting of the shell. Because of these acoustic behaviors, MNBs have been extensively used as UCAs and have shown increasing potential for drug delivery. Under low-intensity ultrasound irradiation, MNBs can be used as contrast agents in ultrasound imaging for EPR evaluation. Through high-intensity ultrasound-mediated cavitation, EPR mediated therapy can be improved with enhanced drug delivery.

### MNBs and ultrasound response of MNBs

#### MNBs

The materials of the encapsulated gas and shell together determine the physicochemical properties of a bubble. The transport properties of the encapsulated core gas mainly affect the stability of the bubbles. Air and bioinert heavy gases such as sulfur hexafluoride or perfluorocarbons are often used in commercial UCAs found in clinics. One approach to increase the stability of UCAs is to use insoluble gases. For example, perfluorocarbons have a higher resistance to water permeation than to air 92, 93. Recently, bioactive gases that have significant therapeutic potential, including nitric oxide 94, 95 and oxygen 96, 97; have been encapsulated in UCAs for parenteral delivery.

The performance of MNBs primarily depends on the physicochemical properties of the shell material. Proteins, polymers, and lipids are mainly used as shell materials for bubbles. The echogenicity of MBs and NBs is significantly different because of the size difference. MBs possess good echogenicity and have been widely used for ultrasound imaging. A reduction in the size reduces the acoustic response of the bubble 98. The microbubbles may be more useful in inducing vessel permeability than nanobubbles due to their larger volume change and higher acoustic response under the ultrasound radiation. The echogenicity of NBs can be improved* via* shell modification and bubble aggregation. Research has shown that NBs are more stable and exhibit a longer circulation time than MBs 99. However, extravasation is different in MBs and NBs. The size of MBs is usually in the order of a few microns. MBs are used as blood pool contrast agents because they are transported after intravenous injection into the bloodstream 100, where they either remain in circulation until taken up by the spleen or the liver or are dissolved over short periods. Typically, tumor vessels are permeable to particles smaller than 1 μm 101. Based on these premises, MBs cannot escape the capillaries and enter the defective tumor microcirculation *via* the EPR effect. Compared with MBs, NBs can penetrate the sites where tumor vascular leakage takes place *via* EPR and can accumulate in the tumor. The gaps between the endothelial cells in tumors, therefore, allow passive targeting with NBs. Fluorescent dye-labeled NBs have been found in the intracellular spaces of tumor cells 99. NBs are also more stable and exhibit longer circulation time than MBs, which leads to further accumulation in tumors. *In vivo* studies have confirmed the ability of NBs to passively target tumor tissues *via* the EPR effect 99. The accumulation of NBs in the tumor area can lead to higher quality imaging and more effective drug delivery. Cavalli et al. showed that amphiphilic drugs and negatively charged complexes can be simultaneously loaded into a NB, which indicates their theranostic capability 102.

#### Vascular permeability enhancement through ultrasound-mediated MNBs

The mechanical effects that ultrasound-mediated MNBs have on the EPR are associated with the process of cavitation, which is mainly due to the oscillation of gas-filled bubbles in an acoustic field. MNBs have different responses to external stimulation based on the features of the applied ultrasound, as shown in Section 2 in the review. Under diagnostic frequency and low sound pressure (<0.1 MPa), bubbles expand and contract with a small amplitude, and the generated energy is low enough to form an SC. This regular oscillation can echo the ultrasonic wave utilized by ultrasound imaging. The SC can create a circulating flow of fluid around the bubble, which is known as microstreaming. Under higher sound pressures (>0.1 MPa), the oscillation of the bubble becomes nonlinear and the expanded diameter is much larger, causing the microbubbles to oscillate drastically. When the sound pressure exceeds a certain threshold, the bubbles instantaneously collapse to release strong jets, shock waves, and shear force (IC). Both SC and IC lead to the creation of pores in the cell membrane and the opening of interendothelial junctions, thus enhancing vessel permeability and improving the extravasation of co-administered drugs 38.

Inducing cavitation *via* MNBs changes the vascular and cell membrane permeability of a tumor. Both SC and IC can cause mechanical disturbance to the vessel walls and cell membranes in cancer tissue, thus improving the EPR effects in the tumor. The application of ultrasound to vessels containing MNBs can change the permeability of the blood vessel wall, resulting in the extravasation of drugs into the interstitial space. The change in the permeability of the capillaries depends on various factors, including shell composition, bubble size, the ratio of capillary diameter to bubble diameter, and ultrasound parameters. In addition to changing the permeability of the blood-vessel wall, the cavitation of MNBs can enhance the permeability of a cell membrane. The collapse of a bubble and the associated production of a jet can instantly break adjacent cell membranes. Small holes are produced within a cell membrane, resulting in either repairable or non-repairable sonoporation. With different ultrasound parameters, short-lived pores are produced within the cell membrane and exogenous materials can thus be transported into the cytoplasm. The collapse of MNBs can also cause cell death in the tumor tissue, which further alleviates solid stress and can reduce the barriers to deeper penetration. Studies have shown that cavitation effects can alter vascular and cell membrane permeability through three different mechanisms: (1) During the regular mechanical disturbance of the oscillating bubbles undergoing SC, the cell membrane potential is changed to promote endocytic uptake 103, 104. (2) The volume of the oscillating bubble changes during the transition from SC to IC. The gap between the vascular endothelial cells is temporarily increased and the integrity of the vascular endothelium is destroyed, thereby allowing enhanced diffusion of the active substances, which can then enter the tissue 71, 105, 106. (3) Based on the sonoporation produced by IC, transient pores are generated within the vascular endothelial cells, which promote the uptake of intracellular macromolecules 86, 107-109.

Cavitation is directly affected by various factors, such as ultrasound frequency, intensity, and the length of US pulses, which in turn influence the EPR in the biological tissues or cells. Low-frequency ultrasound (20-100 kHz) can penetrate the tissues deeper than high-frequency ultrasound (1-3 MHz). Transdermal transport and cell penetration are enhanced as the US frequency decreases 110, 111. Yang et al. 112 experimentally demonstrated the cavitation of polymer shelled MBs mediated by ultrasound fields of different frequencies and sound pressures. MBs loaded with Fe_3_O_4_ nanoparticles ruptured and released the nanoparticles into the cells under exposure to ultrasound. The results showed that cells internalized the Fe_3_O_4_ nanoparticles released by the MBs most efficiently at 1 MHz and 0.25 MPa. The pores in the cell membrane can be simultaneously repaired by the lysosomal membrane associated protein (LAMP-1). When the sound pressure exceeded 0.25 MPa, the severe nonlinear oscillation of the microbubbles may have caused permanent damage to the cells. It has been found that high-intensity focused ultrasound combined with microbubbles can open the blood-brain barrier (BBB), which provides a means for treating brain tumors. Pulsed US causes less negative biological interactions with healthy cells and tissues not intended for treatment 113. For medical purposes, the use of pulsed US is, therefore, more favorable than the use of CW. High-intensity focused ultrasound (HIFU), which can focus US intensity at the target site, has been developed for ablating various types of live tissue 114. This treatment is known to have irreversible thermal effects, including hemostasis and blood vessel occlusion. However, the pulsed-HIFU (pHIFU) treatment can reduce these irreversible thermal effects by reducing the average intensity of US at the target tissue. This pulsed-HIFU (pHIFU) method has been developed to enhance the extravasation and tissue penetration of nanoparticles in the femoral tissue of mice 115.

In addition to cavitation, MNBs can also generate other biological effects, such as thermal effects, under the guidance of high-intensity ultrasonic energy. These biological effects work together to temporarily enhance the permeability of biological tissues and blood vessels, allowing the active substances carried in the MNBs to enter the tumor tissue more easily through the EPR effect, which is the theoretical basis of ultrasound-assisted MNB-enhanced EPR.

### MNB-assisted ultrasonic evaluation of EPR

The high heterogeneity of EPR among individual patients is considered a major barrier to the clinical application of nanomedicines. In preclinical validation, drug delivery systems generally work very well, with significant improvements in both target site accumulation and therapeutic efficacy. Clinically, however, the efficacy of passive tumor-targeted nanomedicines is compromised because of the large inter- and intra-individual variability in EPR. Significant increases in tolerability but hardly any increases in efficacy have been seen in clinical therapy 10, 11, 19. Consequently, it is necessary to develop methods to visualize and characterize the EPR effect before treatment. Based on the assessment of individual EPR, patients presenting with sufficiently high levels of EPR, pre-stratify responders, and non-responders could be pre-selected to individualize and improve nano-chemotherapeutic treatments.

Ultrasound contrast imaging is a simple, non-invasive imaging modality used to evaluate vascular conditions. However, tumors are characterized by tortuous vascularity, angiogenesis, and hypoxia. To address these problems, different strategies for MNB-enhanced ultrasound imaging based on EPR have been developed. One important factor to be considered in ultrasound imaging is the accumulation of MNBs in tumor tissue. As mentioned above, NBs of an appropriate size can penetrate the leakage sites of tumor vascular tissue. EPR-mediated passive targeting can result in the accumulation of NBs in the tumor. However, the enhancement of ultrasound imaging by nanobubbles is reduced relative to microbubbles 115 in clinical applications. Therefore, it is important to maintain its ability to enhance ultrasound imaging while the bubble size is reduced. A sufficient aggregation of NBs within the tumor is beneficial for a high quality image and can be used for enhanced ultrasound imaging of a tumor 116. The shell composition of the bubbles can also be adjusted to increase the echo signals of bubbles in an ultrasonic field. Studies have shown that the incorporation of magnetic nanoparticles into bubble shells can enhance ultrasound imaging 117, 118, which could be used to improve the imaging ability of NBs.

Although MBs cannot pass through blood vessels and enter tumor tissue, efforts can be made to allow them to accumulate around the blood vessels of the tumor. Angiogenesis is a remarkable characteristic in the tumor microenvironment. Endothelial cells and receptors associated with angiogenesis are readily accessible for targeting with MBs. The conjugation of ligands onto the surface of MBs has already been proven successful for MB accumulation in tumors for imaging purposes. It has been reported that a better ultrasound signal was detected from MBs in angiogenic areas after the conjugation of arginylglycylaspartic acid (RGD) peptides onto the MB surfaces 119.

Another important factor in contrast ultrasound imaging for EPR characterization is to identify the correlation between the imaging parameters of tumor blood vessels and drug accumulation in tumors. A US-based assessment of tumor vascularization can potentially be used to predict the efficiency of passive tumor targeting. EPR assessment based on ultrasound imaging is simple, general, and easily transformed for clinical use. In an exemplary preclinical study 28, the relative blood volume (rBV) in tumors was assessed using contrast-enhanced US imaging. The rBV values were correlated with the tumor accumulation of HPMA-based polymeric drug carriers. A good positive correlation was observed, supporting the fact that imaging vascular parameters such as rBV may be useful in predicting EPR-mediated tumor targeting. Applying multi-modal imaging (MRI, μCT, US, and microscopy), Sulheim correlated the accumulation of polystyrene nanoparticles in different tumor models with parameters such as the functionality of tumor vessels measured *via* the inflow of MBs using US 31. The density of functional blood vessels measured by fluorescence microscopy was significantly correlated (p = 0.0056) with nanoparticle accumulation, and the inflow of microbubbles measured with ultrasound also showed a moderate, but significant (p = 0.041), correlation with nanoparticle accumulation, indicating that both the number of vessels and the vessel morphology and perfusion can predict nanoparticle accumulation. This indicates that blood vessel characterization using contrast-enhanced ultrasound imaging or other methods could be valuable in patient stratification for treatment with nanomedicines.

### MNB-assisted ultrasonic enhancement of EPR

MNBs can act as carriers for therapeutic payload delivery. Therapeutic payloads including therapeutic gas, targeting ligands, drug molecules, and nanoparticles can be loaded into the bubble.

The delivery of drugs used for chemotherapy suffers from many problems including severe side effects and the resistance of tumor cells 120. MNBs can be developed as multifunctional carriers for drug transport and delivery. The drugs encapsulated in MNBs are protected from intracellular reactions. MNBs can mediate drug delivery in tumors through EPR effects or active targeting. Particularly, when combined with ultrasound, drug delivery can be controlled and enhanced in tumors. A fluid flow is created when MNBs undergo oscillation mediated by ultrasound 121. This phenomenon increases the drug convection inside the blood vessels and elevates the accumulation of a drug in the surrounding tumor tissues through the EPR effect, resulting in the increased therapeutic index of a drug 121, 122. Local US application can further induce a transient increase in the endothelial cell membrane permeability to enhance the uptake of therapeutic agents by the target cells. Fan et al. proposed a boron-polymer/microbubble complex (B-MB)-assisted focused ultrasound (FUS) treatment for brain glioma 123. B-MBs can simultaneously achieve the safe opening of the blood-brain tumor barrier and enhance boron drug delivery into the tumor tissue using FUS sonication. Cao et al. reported the release of an anticancer drug (DOX) from phase-changeable nanodroplets triggered by low-intensity FUS (LIFU) 124. They found that the intratumoral accumulation and distribution of a drug with LIFU exposure was significantly enhanced. The combination of ultrasound, microbubbles, and gemcitabine is now under phase-II clinical trials for the treatment of pancreatic cancer 125.

Nanoparticles (NPs) such as gold and superparamagnetic iron oxide NPs are widely used in tumor diagnosis and treatment. The delivery of these NPs at the tumor-specific site is a challenging task. Small nanoparticles (<5 nm) can be rapidly removed from vessels by renal clearance or uptake by the liver, whereas large particles (>200 nm) are removed by the spleen or the reticuloendothelial system (RES) 126, 127. NPs can be delivered to specific cancer tissue only by designing a proper carrier system and avoiding the delivery of the drug to normal tissues. MNBs can be used as carriers to deliver NPs to the tumor tissue site* via* the enhanced EPR effect. NPs are often loaded into the shell of MNBs and delivered to tumor cells in a process controlled by ultrasound. A typical example is the delivery of superparamagnetic iron oxide (SPIO) NPs through MNBs. Different types of bubble related carriers including magnetic microbubbles 128 and magnetic nanoliposomes 129 have been developed for *in situ* microbubble generation. Combined with US, SPIO NPs were locally released at the tumor and taken up by the tumor cells though EPR effects. These bubble-based magnetic carriers offer an attractive possibility for clinical MR imaging, hyperthermia, and the magnetically targeted release of therapeutic agents for cancer.

Gaseous molecules including oxygen (O_2_), nitric oxide (NO), hydrogen sulfide, and carbon monoxide have important physiological roles or can elicit biological responses. Therefore, they have significant therapeutic potential 130. These bioactive gases are much smaller than classical drugs and can easily diffuse across the barriers within vessels and cell membranes 130. While the therapeutic effect of a gas is concentration dependent, a major hurdle in biomedical application is the controlled and site-specific delivery of the gas. Compared with solid carriers, MNBs are good containers for gas, with effective loading and controlled release. It is estimated that at least 50-60% of advanced solid tumors contain hypoxic tissue, which is a significant factor leading to the resistance of tumors to treatment 131. Vaupel et al. developed O_2_ nanobubbles for spontaneous oxygenation in response to the tumor microenvironment 131. The intratumoral O_2_ level can be significantly increased by the O_2_ released from O_2_ nanobubbles that have been passively accumulated within the tumor because of the EPR effect. NO plays a critical role in vascular physiology and cancer biology. At low concentrations, NO promotes tumor cell growth by stimulating angiogenesis, while at high concentrations, NO is cytotoxic and may be a useful chemotherapeutic agent 132. MNBs can protect NO from endogenous scavengers and provide an opportunity for targeted delivery, thus providing a therapeutic potential for cancer.

## Advanced ultrasonic carriers and manipulation

MNBs can effectively enhance the ultrasound echo signals for evaluating the EPR effect of a tumor; at the same time, modifications can be made to the membrane shell structure, allowing the easy loading or coupling of other contrast agents, drugs, and targets. Mediated by an ultrasonic field, MNBs can also lead to various useful biological effects such as cavitation and hyperthermia to enhance EPR and achieve a synergistic interaction. Therefore, studies on MNB-based theranostic drug-loading systems have received extensive attention in recent years.

### Adjustable size modulation strategy

The EPR effect is strongly dependent on the size of nanomedicines. If the nanomedicines are too large, they will not be able to leak through the tumor blood vessels into the solid tumor. If they ate too small, they will be quickly removed by the kidneys. Even if the nanoparticles reach the tumor tissue through the blood vessels, the difference in size affects their ability to spread further. Therefore, the design of a drug carrier with adjustable size can promote the penetration and enrichment of a drug inside a tumor. The special properties by which MNBs respond to an external field can be used to change the size of MNBs under the guidance of an external field such as ultrasound and temperature. This can be achieved by devising ingenious designs of the membrane and the gas core component, thus forming an effective diagnostic and therapeutic carrier. At present, two strategies are applied for size adjustment of MNBs: one is "small to large" and the other is "large to small".

**Figure 4:**
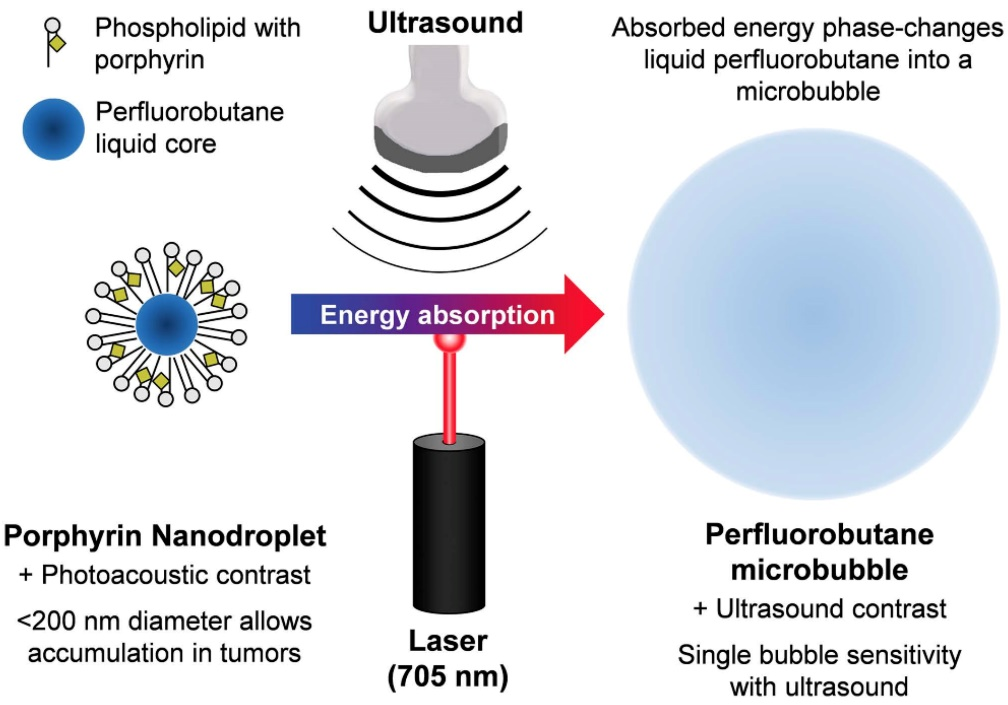
** The “small to large” strategy.** Schematic of the method used to phase-change porphyrin nanodroplets into microbubbles, and the imaging contrast provided by both agents. Porphyrin nanodroplets, which provide photoacoustic contrast because of the strong optical absorption of porphyrin, can absorb acoustic or photonic energy which causes the liquid perfluorobutane core to phase-change into a gas microbubble, thereby providing ultrasound contrast due to its hyper-echogenic and nonlinear acoustic properties 135.

The “small to large” strategy is mainly used in EPR targeting to overcome the contradiction between particle size and the sound intensity of the contrast agent. Nanobubbles exhibit *in vivo* stability and can even reach the interstitial space through the vascular endothelium, providing enhanced tissue imaging and obtaining more abundant imaging information. Thus, nanobubbles are useful to evaluate the EPR effect. However, the clinical practicality of using ultrasound imaging with small-sized bubbles is considerably lower 133. It is, therefore, important to maintain the imaging ability when the bubble size is reduced. One typical class of “small to large” bubbles are phase-inversion UCAs, as seen in Figure 4 134, 135. Nanoparticles or nanodroplets containing liquid fluorocarbons have been extensively explored and designed. The nanoscale and internal liquid environment promotes prolonged stability and tissue penetration. When the external temperature changes under the trigger of ultrasonic energy, the liquid fluorocarbon inside undergoes a liquid-to-gas phase transition and the volume increases accordingly, resulting in enhanced ultrasonic imaging at the lesion site. Finally, the bubble bursts, and the drug that is released in the target region may exert a further synergistic therapeutic effect. Min et al. developed tumor-homing echogenic chitosan-based nanoparticles, in which bioinert perfluoropentane (PFP) was encapsulated into glycol chitosan nanoparticles 136. These particles showed prolonged echogenicity *via* the sustained microbubble formation process in the liquid-phase PFP at body temperature. A significant increase was found in the tumor-homing ability through the EPR effect of the nanoparticles, and this was used for cancer theranostics. Liu et al. prepared folic-acid-modified nanodroplets (FA-NDs) with phase-change ability, which undergo a liquid-gas phase transition under LIFU irradiation 137. FA-NDs were found to be enriched in the tumor area under LIFU, and the resulting micron-sized MBs could enhance the ultrasound imaging signals.

**Figure 5:**
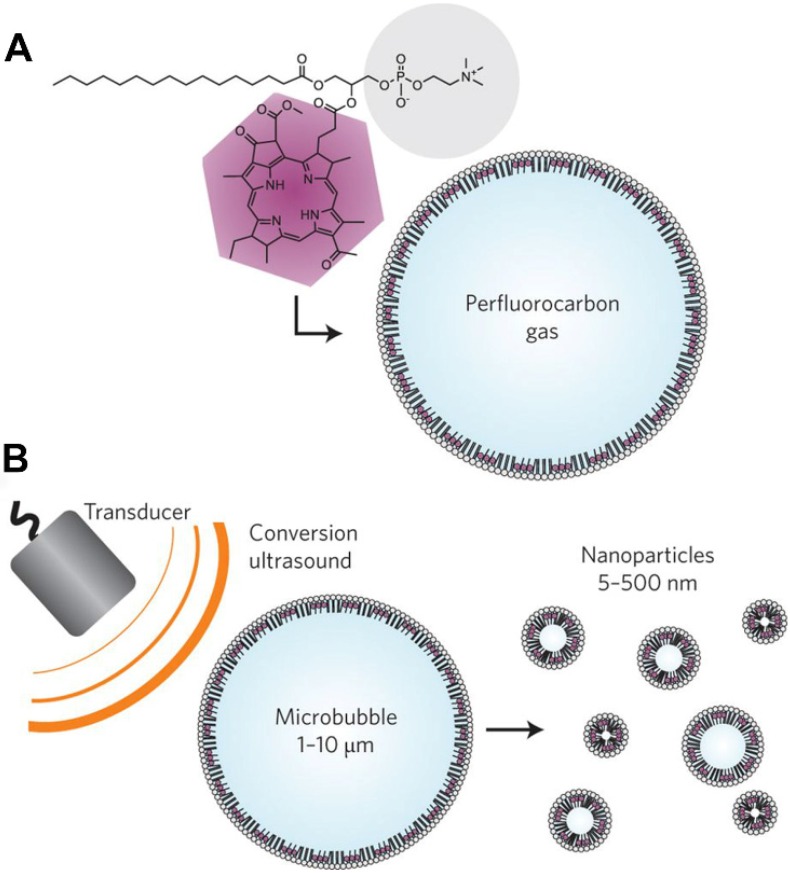
** The "big to small" strategy.** Schematics of porphyrin microbubbles (pMBs) and their micro-to-nano conversion. A. The pMBs consist of a BChl-lipid shell encapsulating perfluorocarbon gas. B. Conversion of pMBs to porphyrin nanoparticles (pNPs) via sonication with low-frequency, high-duty-cycle ultrasound (conversion ultrasound) 138.

The "big to small" strategy is mainly used to help microbubbles bypass the EPR effect and release drugs at the tumor site. Micro-sized bubbles are good angiographic contrast agents and drug carriers, but they cannot penetrate blood vessels to reach the tissues. If MBs can somehow be made smaller to become nanobubbles after reaching the tumor blood vessels and completing the imaging function, they could spread across the vascular barrier and into the tumor tissue to be used for further tissue imaging and drug release. Huynh et al. reported a method used for the conversion of microbubbles to nanoparticles using low-frequency ultrasound, as shown in Figure 5 138. Porphyrin loaded microbubbles can be used as tri-modality contrast agents for ultrasound, photoacoustic, and fluorescent imaging. During exposure to ultrasound, the microbubbles burst and formed smaller nanoparticles, which maintains the imaging and therapeutic properties and allows drug delivery to the tumor. With this conversion, microbubbles can potentially be used to bypass the EPR effect when delivering drugs to tumors.

Therefore, through the delicate design of the bubble structure and the appropriate introduction of external triggers, a new size-controllable theranostic agent with ultrasound enhancement effect and tissue penetration ability can be constructed to evaluate and enhance the EPR effect.

### *In situ* generation

Different types of stimuli have been reported to trigger the *in-situ* generation of bubbles in a solid tumor. These types of stimuli are usually endogenous and are closely related to changes in the tumor microenvironment. Agents that are responsive to such stimuli are encapsulated in nanocarriers, which accumulate at the tumor site through the EPR effect. Stimuli are then applied, thus triggering the generation of gas free bubbles at the tumor tissue. The advantages of this *in situ* generation strategy are as follows: (1) The bubbles generated *in situ* at the tumor site do not need to be transported through blood. They can, therefore, avoid various biological barriers in the human body and can also bypass the requirement for EPR in changing the bubble size. (2) The generation of bubbles enhances the ultrasound imaging effect at the tumor site, which is conducive to diagnosis. If the generated gas can also be used for treatment, targeting, diagnosis, and treatment can be integrated.

**Figure 6:**
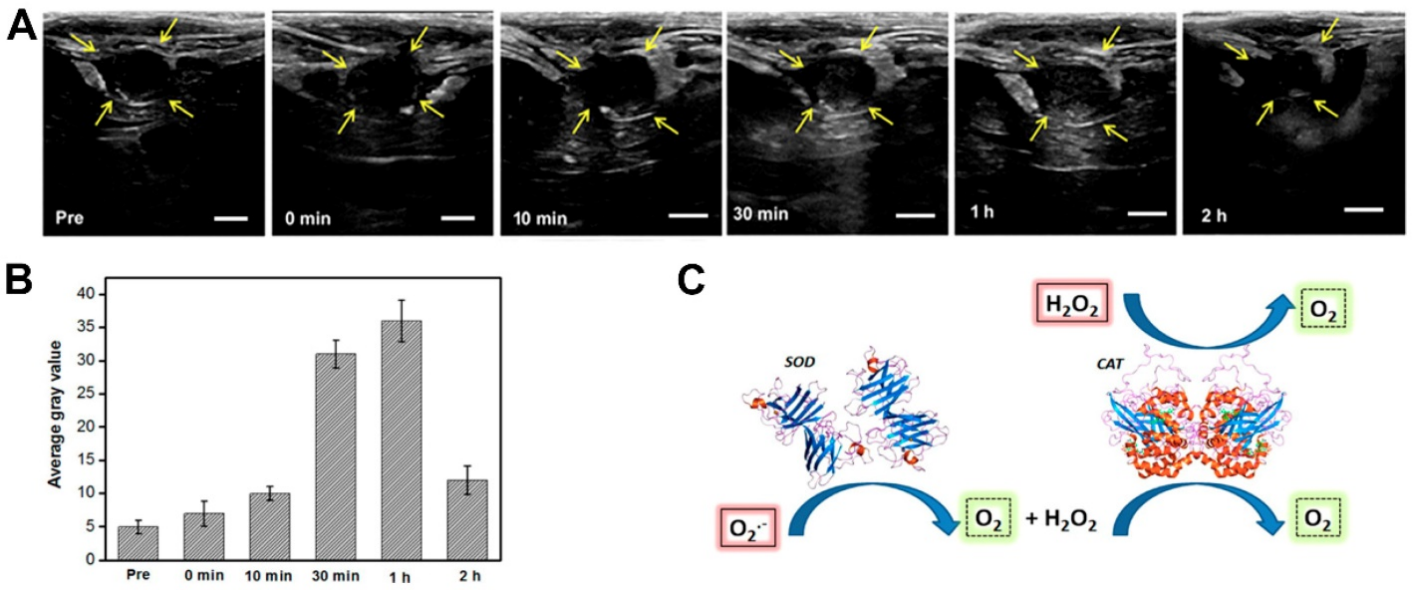
***In vivo* US imaging.** A US imaging and B the corresponding average gray values of VX2 tumors on rabbit livers before and after intravenous injection of SGC (SPIO@GCS/acryl/biotin-CAT/SOD-gel) at various times. C. Schematic mechanism of responsive bubble generation for US imaging. D. Yellow arrows indicate outlines of the VX2 tumors. The bars in A correspond to 0.5 cm. The standard deviation in B is derived from a group within the region of interest (ROI) (n = 3) 141.

For example, the production of reactive oxygen species (ROS) is much higher in cancer tissues than in normal tissues. H_2_O_2_ is an ROS and a diagnostic marker in the development of certain diseases. Studies have found that Prussian blue nanoparticles have a catalase-like function in neutral environments, catalyzing H_2_O_2_ to produce oxygen 139. When the oxygen generated exceeds the saturation concentration, it can form free oxygen bubbles, enhancing ultrasound imaging. Together with the MRI properties of Prussian blue nanoparticles, this could be used as an ultrasound/magnetic resonance bimodal imaging system which is triggered by the disease environment 140. Similarly, Wang et al. 141 constructed a multifunctional carrier system loaded with two catalase enzymes and SPIO. The two enzymes catalyze the reaction of the ROS in the body under pathological conditions to produce oxygen that can be utilized for ultrasound imaging, as can be in Figure 6. The changes to the reaction environment also enhance the MRI capabilities of SPIO within the system.

### Multi-gradient targeting

In the above studies, the aggregation of bubbles and release of drugs in the target region were mainly dependent on various exogenous and endogenous stimuli and were not caused by specific active targeting. EPR and the (over-) expression of angiogenesis-related surface receptors are key features of tumor blood vessels. As a consequence, EPR-mediated passive and ligand-based active targeting have attracted considerable attention. Kunjachan et al. visualized and quantified passive and active tumor targeting using RGD- and NGR-modified polymeric nanomedicines 142. Wu et al. developed paclitaxel-loaded and A10-3.2 aptamer-targeted poly(lactide-co-glycolic acid) nanobubbles, which can be aimed specifically at prostate cancer cells to sustainably release the loaded PTX via the EPR effect and US-triggered drug delivery 143. Li et al. reported the use of neuropeptide Y Y1 receptor-mediated biodegradable photoluminescent nanobubbles as UCAs for targeted breast cancer imaging 144.

Rapid and efficient early binding of MNBs to the blood vessels of the tumor occurred through vascular targeting, but passive targeting was significantly more efficient over time. These results indicate that a combination of passive and active targeting is required for effective tumor imaging and therapy. Based on the above ideas, a multi-gradient targeting strategy has emerged. Multi-gradient targeting strategies refer to the further coupling of specific targeting factors on MNBs to combine active targeting with EPR-mediated passive targeting. When such MNBs are intravenously injected into the body, they become actively enriched in the tumor blood vessels under the action of specific targeting ligands, contributing to clearer ultrasound imaging. Following the application of an ultrasound field or various other internal and external sources of stimulation, the structure and/or the size of the MNBs changes. On the one hand, this change triggers various biological effects, which affect the permeability of the tumor blood vessels and enhance the EPR effect. On the other hand, the drug is released, forming a high drug concentration gradient in the microcirculation of the tumor tissue, efficiently allowing the drug to enter the tumor tissue for treatment by virtue of the EPR effect.

Several classes of ligands have been conjugated onto the surface of UCAs to achieve better targeting of tumor cells, including peptides, aptamers, and affibody molecules. These targeting ligands allow the UCAs to remain at a size at which the EPR effects can be exploited 145. Cell membranes are a new type of shell material that have been used in the construction of bubbles. The inherent properties of natural lipids and protein components on a membrane can be used for natural targeting. For example, platelet membranes are rich in natural vessel adhesive components such as α_2_β_1_, α_5_β_1_, α_6_β_1_, and α_b_β_3_, and the glycoproteins, GPIb-IX-V and GPV, which can naturally target injured vasculature 146. Mingxi Li et al. used platelet membranes to fabricate bio-nanobubbles and demonstrated their lesion-targeting ability 147. The abnormal vasculature within tumors provides the possibility of using bio-shelled bubbles for the targeting of tumors.

Active targeting can be performed by modifying bubble shells using a range of targeting ligands; such bubbles can therefore be applied in different types of cancer. There are several strategies that allow the binding of ligands to the bubble shell. First, avidin or streptavidin is commonly used for non-covalent attachment of biotinylated ligands onto the shell materials 148. Yang et al. used the avidin-biotin method to combine NBs with biotinylated anti-ErbB2 Affibody® molecules 149. The resulting NB-Affibody had a specific affinity for human epidermal growth factor receptor type 2 (HER2)-over-expressing tumors. Second, the covalent binding of ligands to the shell has also been explored. One approach involves the binding of activated carboxylic groups to shells with amino-groups from the lysine residues of binding ligands 145. In a third approach, the ligands are coupled with the shell components (e.g., to phospholipids), followed by the formation of MBs. Chung-Hsin Wang et al. conjugated sgc8c aptamers to functional lipids and prepared DOX-loaded acoustic droplets 150. The fully vaporized droplets resulted in the highest DOX uptake by cancer cells.

**Figure 7:**
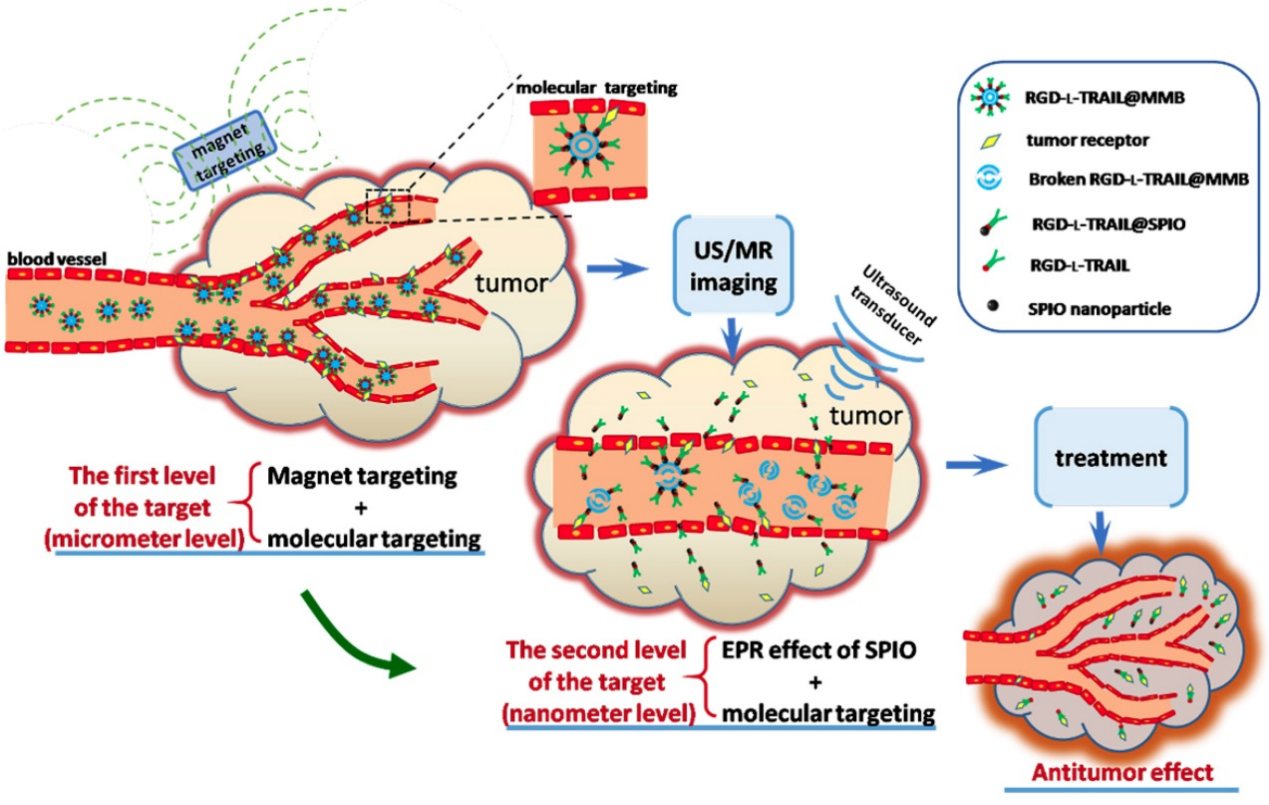
Schematic diagram showing multi-gradient targeting strategy of RGD-l-TRAIL@MMBs for tumor diagnostics and therapy 128.

Multi-gradient targeting can be applied to both microscale and nanoscale bubbles. Nanoscale bubbles modified with targeting ligands are good carriers. Li et al. reported that the soft biodegradable glycine/PEG/RGD-modified poly (methacrylic acid) nanobubbles can be applied as intelligent theranostic vehicles for drug delivery 144. Gao et al. developed the targeted ultrasound-triggered phase transition nanodroplets for Her2-overexpressing breast cancer diagnosis and gene transfection 151. For micron-sized bubbles, Duan et al. 128 demonstrated a multi-gradient continuous targeting strategy based on the RGD-l-TRAIL-labeled magnetic microbubbles shown in Figure 7 for cancer therapy. The designed RGD-l-TRAIL@MMB system had a complex but elaborate multilayer structure: gas in the core for US imaging, SPIO on the shell for MRI imaging and magnetic targeting control, and RGD-l-TRAIL ligands for an enhanced combination of the tumor-targeted delivery of TRAIL. First, the RGD-l-TRAIL protein on the MMB surface enables the microbubbles to actively target angiogenesis of a tumor. The size of the micro-scaled bubbles around the tumor is beneficial for real-time US imaging and MRI to clearly delineate the tumor margin. Therefore, it is micron-scale targeting and diagnosis. Second, after US imaging, the microbubbles *in situ* around the tumor rupture and the nanoscale SPIOs with associated RGD-l-TRAIL molecules can travel through the blood vessels and enter the tumor tissue because of the tumor ligand induced integrin αvβ3-receptor-mediated endocytosis. At this stage, the tumor tissue can also be imaged using MRI. With the accumulation of RGD-l-TRAIL and SPIOs in the tumor tissue and cells, tumor apoptosis induced by TRAIL molecules can also be observed during this process; therefore, this process involves nanoscale targeting and diagnosis. The multi-gradient targeting drug delivery system has a reasonable structure, an adjustable drug dosage, and a clear target. It is a typical example of applying a multi-gradient targeting strategy for *in vivo* diagnosis and treatment.

### Multi-field association

In addition to the ultrasound field, external factors including light, magnetic fields, radiation, and microwaves affect EPR. Internal factors such as pH, high concentrations of active oxygen, and various special cytokines are provided by the special pathological environment of the lesion. The mechanism by which each factor affects the EPR is different. Combining multiple energy fields can exert the combined effects of all of the different mechanisms to obtain an optimized and enhanced EPR effect.

Local heating of the tumor site tends to increase the accumulation of nanomedicine, resulting in more effective intratumoral drug delivery. Hyperthermia enhances the increase in nano drug delivery within tumors because of the increased blood flow to the tumor caused by vasodilation. This increased vascular permeability is due to the increased intercellular space between endothelial cells 152-154. Through the photothermal conversion effect, the irradiation of a tumor with a laser can also change the physiological state of the tumor and enhance the penetration of the nano drug into the tumor. By constructing a versatile theranostics carrier that responds to light and other external fields, thermal effects can be combined with other effects to improve vascular permeability. Ke et al. 155 constructed a multifunctional nanocapsule (NC) by loading perfluorooctylbromide (PFOB) and superparamagnetic iron oxide nanoparticles into poly(lactic acid) (PLA) NCs, followed by the formation of PEGylated gold nanoshells on the surface. Such a theranostic agent could efficiently kill tumor cells using NIR laser irradiation under the guidance of contrast-enhanced US/MRI to achieve great therapeutic effectiveness without systemic toxicity to the patient. Above all, the bimodal imaging guidance could display dynamic complementary information about a tumor to help alter the treatment on an individual basis with high efficiency.

**Figure 8:**
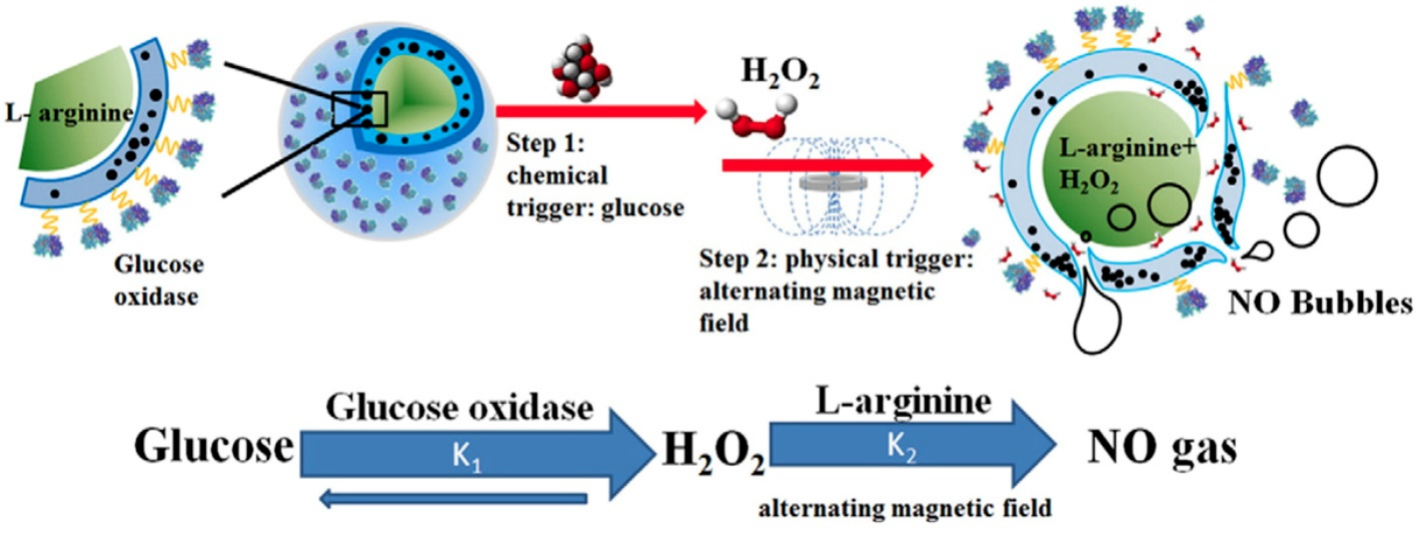
Schematic diagram of microvesicles encapsulated with magnetic nanoparticles and glucose oxidase for the dual-stimuli responsive programmable delivery model. The encapsulated glucose-specific enzyme catalyzes glucose into gluconic acid and H_2_O_2_. The subsequent alternating magnetic field increases the porosity of the polymer shell, leading to the reaction between L-arginine and H_2_O_2_ to produce nitric oxides 157.

The strategy of combining internal and external stimuli is also beneficial. Yang et al. 156, 157 constructed a composite microcapsule with internally encapsulated L-arginine, SPIO in the membrane shell, and glucose oxidase on the surface, which is a double-response, microcapsule transport system that reacts to both glucose and external magnetic fields, as shown in Figure 8. First, the glucose oxidase on the surface of the microcapsule specifically reacts with glucose in diabetic patients, reducing the glucose concentration and generating H_2_O_2_ and gluconic acid. The magnetic nanoparticles in the microcapsule shell then move under the action of the external magnetic field to generate gaps in the membrane, and the internally loaded L-arginine further reacts with the H_2_O_2_ to form NO. Therefore, it is apparent that through the dual response of the microcapsule structure to glucose and magnetic fields, high concentrations of glucose can be reduced and the generated NO gas can enhance the ultrasound imaging and the EPR effect, thus treating the disease. In addition to responding to external magnetic fields, the SPIO in the system also functions for MRI imaging. This system can be used as a multifunctional carrier platform for the effective regulation of blood glucose.

**Figure 9:**
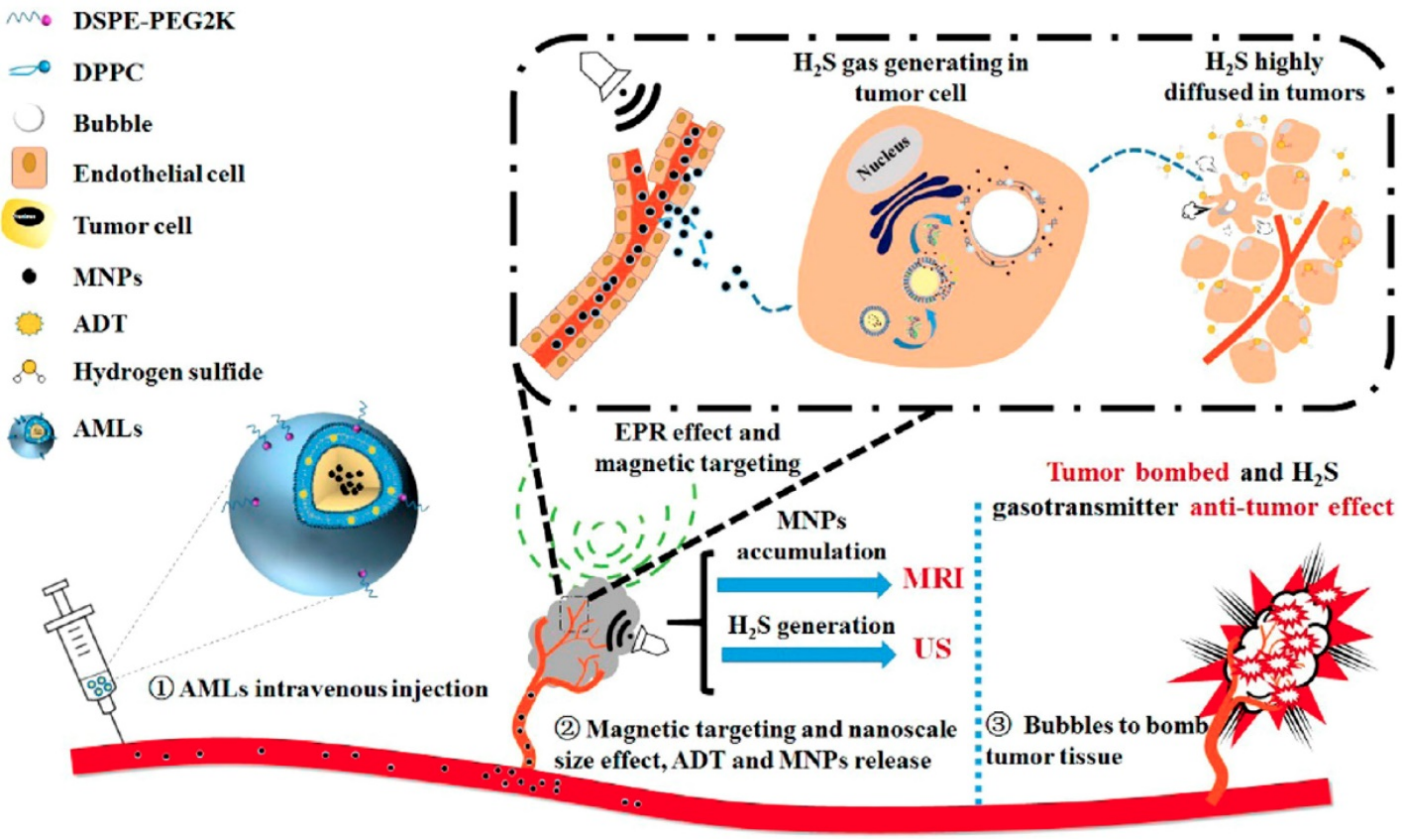
Concepts and schematics of AMLs and their nano to micro conversion for US/MR dual modal imaging and the spatiotemporal-bombed combination tumor accurate therapy 129.

Liu Yang et al. 129 designed a liposome drug delivery system, as seen in Figure 9, which is a complex structure of a therapeutic gas prodrug, anthranil trisulfide, and magnetic nanoparticles, which is efficiently concentrated at the targeted tumor site by the EPR effect under the control of an external magnetic field. Under the catalysis of biological enzymes such as CBS and CSE in the tumor, hydrogen sulfide is produced, which has antitumor properties. The liposome-loaded magnetic nanoparticles respond specifically to the external magnetic field. The intratumoral targeted aggregation of liposome can monitored and regulated by MRI; and the hydrogen sulfide microbubbles catalyzed by the enzyme enhance the intratumoral ultrasound contrast, leading to a greater intensity of ultrasound energy. The microbubble then ruptures, ultrasonic cavitation leads to physical tumor ablation, and the antitumor properties of the hydrogen sulfide itself lead to necrosis of the original dense intratumoral tissue, creating favorable conditions for the use of nanocarriers. This liposome drug system can achieve better theranostic destruction of a tumor under ultrasound/magnetic resonance bimodal image monitoring.

There are also multifunctional carrier systems with sophisticated designs that combine lasers, heat, radiation, and other external and ultrasonic fields to improve the permeability of tumor blood vessels and tissues and deliver drugs effectively.

**Figure 10:**
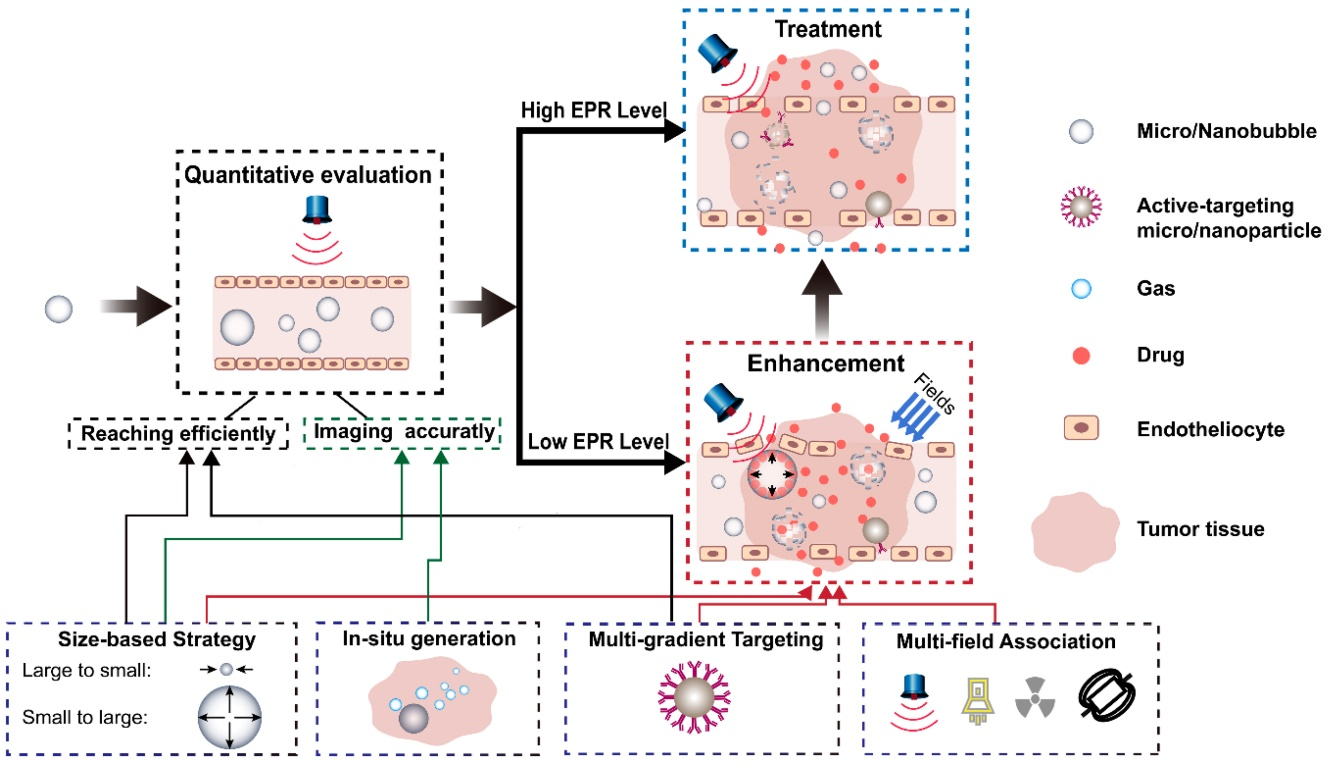
Ideal process for tumor diagnosis and treatment based on the EPR effect by ultrasound and its contrast agents.

The ideal process for tumor diagnosis and treatment based on the EPR effect is shown in Figure 10. Before starting treatment, an accurate individualized assessment of the EPR level should be performed for each individual patient, and different treatment options should be developed based on the results of the assessment. For patients with high EPR levels, an appropriate drug delivery system can be selected for treatment. For patients with low EPR levels, the EPR effect should be enhanced using various physical and pharmacological methods before treatment. Would it be possible to construct a multifunctional theranostics carrier that can play a role in all aspects of the evaluation, enhancement, and treatment, realizing the integration of EPR evaluation, enhancement, and EPR-based tumor treatments on a single platform? MNB-based drug delivery systems have become a potential choice because of their special properties such as imaging, drug release, changing structures, and production of various biological effects that can be mediated with ultrasound fields.

Around the process of tumor diagnosis and treatment and applying the four strategies mentioned, the potential of MNBs can be described in detail, as seen below in Figure 10. Firstly, MNBs can be used as contrast enhancers to visually and quantitatively evaluate the EPR levels of a patient. The accuracy of the assessment depends on two key processes: (1) Whether MNBs can efficiently reach tumor blood vessels through blood circulation, and (2) whether a strong echo signal can be generated after reaching the tumor site. The various strategies mentioned can have a positive effect on both of these processes. As described previously, the "small to large" size control strategy has been applied to construct small-sized nanodroplets that have higher stability in the blood circulation than MNBs. A multi-level targeting strategy can also be applied to couple the targeting factors on the surface of the nanodroplets to reach the tumor blood vessels more efficiently. In the latter process, through the mediation of an external energy field, the liquid droplets within the nano-droplet core can be transformed, thus increasing the volume to generate a stronger echo signal which is beneficial to ultrasound imaging. It is also possible to use the "*in situ* generation" strategy to generate bubbles *in situ* using the specific microenvironment of the tumor to create ultrasound contrast with which to evaluate the EPR.

Second, for patients with low EPR levels, MNBs can be combined with internal biochemical triggers and external energy fields to enhance the EPR effect using various strategies, as shown in Figure 10. For example, the "large to small" size control strategy facilitates the micron-sized bubbles to bypass the EPR effect and become smaller nanobubbles that can penetrate the vascular endothelial cell gap, allowing movement into the tumor tissue. The “multi-field association” strategy comprehensively uses light, heat, ultrasound, radiation, and various endogenous factors to increase vascular permeability and enhance the ability of the drug components carried by MNBs to spread out of the blood vessels. The “multi-gradient targeting” strategy allows the drug released after the rupture of a bubble to utilize molecular targeting to efficiently reach the tumor tissue through the blood vessel.

In addition, while enhancing the EPR effect, various energy fields can mediate the gradual disintegration of the structure of MNBs, releasing the various active substances the MNBs carry, including drugs, other nanoparticles, and therapeutic gases. These active substances are enriched in tumor tissues by the enhanced EPR effect and lead to further therapeutic or real-time imaging functions.

## Summary and future perspectives

In the complex and variable micro-environment of a living organism, the key to effective treatment is drug components reaching a lesion efficiently and exerting continuous therapeutic effect. The nano carrier targeted drug delivery system, based on EPR, is one way to effectively improve the anticancer effect of drugs. In recent decades, various forms of multifunctional carrier materials such as liposomes, MNBs, micelles, nanoemulsions, magnetic nanoparticles, polymer nanoparticles, and dendrimers have been designed and developed to improve the aggregation and permeability of the drug at the tumor site. In this review, we focused on the effects of ultrasound and MNBs on EPR, discussed in detail the potential mechanisms by which their respective roles and synergies affect EPR, and illustrated new strategies that have been developed to enhance EPR. Although these new strategies do show promise, further research is needed in this area to demonstrate effective drug delivery and therapeutic effects. In this study, we discuss the advantages of MNB-assisted ultrasound to enhance the EPR effect, the current problems, and any possible countermeasures.

### Advantages

MNBs are distinctly distinguished from other carrier materials in that they have multiple forms of response to the ultrasound field, and the rich biological effects of these responses have a potential role in both EPR imaging and enhancement processes. Under ultrasound energy mediation, the MNB theranostic carrier can affect the EPR in combination with various favorable conditions. First, ultrasound and MNBs can both affect the EPR. The ultrasonic streaming and hyperthermia effect from the ultrasound itself can improve the ability of a drug to diffuse into a tissue, promote the extravasation of drugs, and enhance the permeability of blood vessels. MNBs can also be used as drug carriers with certain EPR effects using various strategies such as structural design, size control, and modification of the membrane shell. Second, ultrasound can be used to further assist MNBs, which are imaged by low-intensity ultrasound energy to evaluate EPR. MNBs can also rupture under high-intensity ultrasound energy and produce biological effects such as cavitation, which leads to the more efficient transport of a drug into the tumor tissue through the vascular endothelial cells. Through sophisticated design, the internal and external source energy fields can be combined, making full use of “adjustable size modulation”, “*in situ* generation”, “multi-gradient targeting”, and “multi-field association” strategies, which can further improve the penetration ability and delivery efficiency of drugs at the tumor site.

### Challenges and countermeasures

#### 5.2.1 Size barrier

The size of the MNBs is still a barrier to the EPR effect. The efficacy of a diagnostic and therapeutic agent through the EPR effect is size-dependent. Related research has shown that liposomes with a hydrodynamic particle size between 100 and 200 nm showed a 4-fold higher accumulation in tumors than liposomes with a particle size of less than 50 nm and above 300 nm 158. The preparation of nanobubbles with a size of less than 200 nm remains challenging. Although studies have shown that ultrasound can rupture lipid microbubbles in the body and form smaller nanoparticles with the same optical properties as the original microbubbles, meaning that micron-sized bubbles can bypass the EPR effect when delivering drugs to the tumor, there is still a need to develop small-sized nanobubbles with the ability of enhanced accumulation. Therefore, it is critical that new preparation processes are developed, and that various adverse problems such as the weak imaging effects caused by small-sized nanobubbles and the decreased drug-loading capacity are dealt with. The combination of NBs with other nanocarriers is one possible way to increase the efficiency of these bubbles in terms of drug delivery.

#### Structural complexity

Multi-modal imaging, in which targeting factors are coupled with the response to various energy fields and other functions, makes the structure of MNBs complex, thus increasing the difficulty in constructing carriers. Therefore, it is necessary to further study and optimize the preparation method of MNBs theranostics carriers, improve the fineness, controllability, and repeatability, and finally to achieve standardization. It is also necessary to further study the effects of MNB carrier structures on their function, such as the material from which membrane shells are built, size, drug-loading mode, and the effects of target type on imaging and targeting. In addition, in the complex and variable microenvironment of the body, it is very challenging to simultaneously achieve a targeted system with high diagnostic sensitivity, high specificity, and good therapeutic effect. The more complex a structure is, the more risk there is, so it is difficult to anticipate the realization of a function and to achieve clinical transformation, especially when using relatively large targeting systems (hundred nanometers and above) such as MNBs. The sensitivity of different biological tissues to ultrasound is quite different, so the applied ultrasound parameters will need to be changed accordingly. The ultrasound parameters used to stimulate the MNBs would also need to be adjusted for use with the different kinds of bubbles.

More *in vivo* experiments are needed to clarify the toxicity, distribution, and pharmacokinetics of a carrier within the body and to provide abundant evidence for clinical application. It is foreseeable that with the continuous development of biomedical nanotechnology based on ultrasound and MNBs, more innovative and more powerful theranostics carriers will be designed and developed to further promote tumor-targeted therapy.

#### Potential safety concerns

One of the important principles of drug delivery system development is safety. The safety issues for micro/nano-bubble-assisted ultrasound to enhance EPR effects, such as micro/nano-bubble toxicity 159-161 and damage to vessels through acoustic energy still need to be fully investigated on priority for future clinical applications 162, 163. The safety issues related to ultrasound have been addressed in Section 2 of this review. The biodegradability and metabolites of ultrasound contrast agents should be investigated. Although some studies have investigated the short-time toxicity, safety tests for biocompatibility, acute toxicity, subacute toxicity, carcinogenicity, developmental toxicity, immunotoxicology, genotoxicity, and irritation to blood vessel should be conducted in the future, especially when MNBs are coupled with ultrasound energy.

## Figures and Tables

**Table 1:** Summary of ultrasound classification and acoustic vehicles for tumor theranostics.

Payload classification	Ultrasound Classification	Therapeutic Substance	Vehicle	Tumor Type	Author, Year, Publishing Periodicals, Ref.
Chemicals	Low-frequency Ultrasound	Paclitaxel (PTX)	poly(lactide-co-glycolic acid) nanobubbles	prostate cancer	Wu, 2017, Int J Nanomedicine, 143
		Endostar	lipid microbubbles	colon cancer	Zhang, 2014, Cancer Lett, 164
	High-frequency Ultrasound	Cilengitide nanoparticles (CGT-NP)	phospholipid-based microbubbles	gliomas	Zhao, 2016, J Controlled Release, 165
	Focused Ultrasound	boron drug	polymer microbubbles	brain glioma	Fan , 2019, ACS Appl Mater Interfaces, 123
		Doxorubicin (DOX)	phase-changeable nanodroplets	breast cancer	Cao, 2018, Theranostics, 124
		Doxorubicin(DOX)	lipid microbubbles	brain tumor	Park, 2017, J Control Release, 166
Nanoparticles (NPs)	Low-frequency Ultrasound	Gold nanoparticles and Gemcitabine and miR-21 Inhibitor	Dendrimer-Entrapped Gold Nanoparticles	pancreatic cancer	Lin, 2018, Theranostics, 167
	High-frequency Ultrasound	superparamagnetic nanoparticles (MNPs) and Anethole dithiolethione (ADT)	magnetic nanoliposome (AML)	HepG2	Liu ,2017, ACS nano, 129
		superparamagnetic iron oxide nanoparticles (SPIO) and RGD‐l‐TRAIL	magnetic microbubbles (Polymer microbubbles) .	colon cancer	Duan, 2016, Adv Funct Mater, 128
	Focused Ultrasound	superparamagnetic iron oxide nanoparticles (SPIO)- Doxorubicin (DOX)	lipid microbubbles	rat cerebral glioma	Fan , 2016, Theranostics , 168
Therapeutic gases	Low-frequency Ultrasound	O 2, Paclitaxel (PTX)	Lipid Microbubble	ovarian cancer	Liu, 2015 Cancer Lett, 169
		NO/DOX	Ultrasound responsive liposome	breast cancer/ pancreatic cancer	Wang, 2017, J Control Releae 170
		CO_2_	Hollow Mesoporous Silica Nanoparticle	pancreatic cancer	Zhang, 2015, Theranostics, 171
	Focused Ultrasound	NO	Hollow Mesoporous Silica Nanoparticles	pancreatic cancer	Zhang, 2016, Acs Nano, 172

## Abbreviations

EPR: permeability and retention
UCA: Ultrasound contrast agent
TI: thermal index
MI: mechanical index
FDA: Drug Administration
PnP: negative pressure
CW: continuous wave
DOX: Doxorubicin
GNPs: gold nanoparticles
DDS: drug delivery system
PIV: particle image velocimetry
SC: stable cavitation
IC: inertial cavitation
SEM: scanning electron microscopy
AFM: atomic force microscopy
TMC: Transmembrane current
CAD: computer-aided detection
UOM: ultrasound-modulated optical microscopy
CEUS: contrast-enhanced US
IVUS: intravascular ultrasound
BFR: blood flow resistance
MNBs: micro/ nanobubbles
UCAs: ultrasound contrast agents
PLGA: poly-(lactic-co-glycolic acid)
rBV: relative blood volume
BBB: blood-brain barrier
B-MB: boron-polymer/microbubble complex
FUS: focused ultrasound
LIFU: low-intensity focused ultrasound
NPs: nanoparticles
RES: reticuloendothelial system
PFP: perfluoropentane
FA-NDs: folic-acid-modified nanodroplets
pMBs: porphyrin microbubbles
pNPs: porphyrin nanoparticles
ROS: reactive oxygen species
PFOB: perfluorooctylbromide
PLA: poly(lactic acid)
NCs: nanocapsules
PSV: peak systolic velocity
SGC: SPIO@GCS/acryl/biotin-CAT/SOD-gel
PFC: perfluocarbon
AML: the anethole dithiolethiones (ADT)-loaded magnetic nanoliposomes
CBS: cystathionine-*β* synthase
CSE: cystathionine-γ-lyase
MMB: magnetic microbubbles
